# Danger to Laity

**Published:** 1901-08

**Authors:** 


### DANGER TO LAITY.

### Doctors Repudiate Professor Atwater's Theory of Food Value for Alcohol.

TESTIMONY THAT THE TEXT-BOOKS ARE ACCURATE.

The American Medical Association has just held its annual meeting in St. Paul, Minn. During its sessions, the American Medical Temperance Association, composed of eminent physicians and teachers in medical colleges, members of the American Medical Association, always holds one or more meetings for the special purpose of promoting scientific study and investigation into the action of alcohol in health and disease. The meeting this year shows a great advance in the scientific study of alcohol and its action on the body. In the ten years of its existence, its membership has grown to over two hundred, and the number of papers and discussions, all of a scientific and technical character, are increasing, so that literally this is the most authoritative organization studying the alcohol question in this country. Of the ten papers read at the St. Paul meeting, three of them discussed Professor Atwater's experiments and conclusions, then passed the following resolutions as the unanimous opinion of the Association:

"Whereas, The American Medical Temperance Association, the members of which are physicians and medical teachers who have devoted years to the study of alcohol and its effects, and who are conversant with the work done by scientific men the world over to determine the effects of alcohol when given in any quantity, have noted the teaching of Professor W. O. Atwater, of Wesleyan University, upon the food and medical value of alcohol as set forth by him in the pages of the influential lay press. Be it

"Resolved, That this association utterly repudiates the pro-alcoholic doctrine of the said Professor W. O. Atwater as being contrary to the evidence deduced by scientific experimentation, and that his conclusions are unwarranted by the evidence resulting from his own experiments. Be it further

"Resolved, That this association regards the teaching of Professor W. O. Atwater as erroneous, and a source of danger to the laity insomuch as such teaching contributes towards the increased consumption of alcohol beverages by giving supposed reason for their safe use."

(Signed) N. S. Davis, M. D.,
President, Chicago, Ill.
T. D. Crothers, M. D.,
Secretary, Hartford, Conn.

Two other papers pointed out the evils from the use of cigarettes and tobacco on neurotics and young persons. One paper critically reviewed the school-book teachings on alcohol, sustaining their claim to scientific accuracy in nearly all the books used.

The address of both the president and vice-president described the folly of efforts to check disease and degeneracy by ignoring alcohol as one of the active causes, also the conflict of experience with theory and tradition. The other papers read discussed the causes of the popularity of alcohol as a beverage, and its danger in high altitudes; also the substitutes for its use in medicine.

The value and reliability of these papers is evident from the fact that eight of the ten authors are active or emeritus professors in medical colleges. Four of them are medical journalists, two of whom are in active practice.—*Charlotte Medical Journal.*

